# Impact of a web‐based breast cancer surgery decision aid on knowledge and perceptions of feeling informed in clinics that care for socioeconomically disadvantaged patients: An Alliance Clinical Trial (A231701CD)

**DOI:** 10.1002/cncr.70314

**Published:** 2026-02-11

**Authors:** Jessica R. Schumacher, Bret M. Hanlon, David Zahrieh, Paul J. Rathouz, Jennifer L. Tucholka, Grace McKinney, Angelina D. Tan, Catherine R. Breuer, Lisa Bailey, Anna M. Higham, Julie S. Wecsler, Alicia Arnold, Anthony J. Froix, Scott Dull, Andrea M. Abbott, Stephanie G. Fine, Kandace P. McGuire, Anna S. Seydel, Patricia McNamara, Selina Chow, Heather B. Neuman

**Affiliations:** ^1^ University of Wisconsin School of Medicine and Public Health Madison Wisconsin USA; ^2^ University of Wisconsin Carbone Cancer Center Madison Wisconsin USA; ^3^ Alliance Statistics and Data Management Center Mayo Clinic Rochester Minnesota USA; ^4^ University of Texas at Austin Dell Medical School Austin Texas USA; ^5^ Bay Area Tumor Institute Oakland California USA; ^6^ Carle Cancer Center Urbana Illinois USA; ^7^ Stroger Hospital of Cook County Chicago Illinois USA; ^8^ Augusta University Health Augusta Georgia USA; ^9^ Hawaii NCORP University of Hawaii at Manoa Honolulu Hawaii USA; ^10^ Montana Cancer Consortium NCORP Billings Clinic Billings Montana USA; ^11^ Medical University of South Carolina Charleston South Carolina USA; ^12^ University of New Mexico Albuquerque New Mexico USA; ^13^ Virginia Commonwealth University/Massey Cancer Center Richmond Virginia USA; ^14^ Marshfield Clinic Marshfield Wisconsin USA; ^15^ Alliance Protocol Operations Office University of Chicago Chicago Illinois USA

**Keywords:** breast cancer, surgery, engagement, knowledge, decision aid, socioeconomic disadvantage

## Abstract

**Objective:**

To test the effectiveness of a surgical web‐based decision aid (DA) in improving knowledge.

**Summary of Background Data:**

DAs support decision making by providing information about the options.

**Methods:**

A stepped wedge trial was conducted in 10 National Cancer Institute Community Oncology Research Program clinics (Alliance for Clinical Trials in Oncology). Clinics were randomized to time of transition from usual care (UC) to delivery of a web‐based DA. Patients with stage 0 through 3 breast cancer being considered for surgery were enrolled. Knowledge (primary outcome) was measured using the Breast Cancer Surgery Decision Quality Instrument and patients were asked, “How informed do you feel?” Intervention effects were tested with linear mixed‐effects models, accounting for surgeon and clinic‐level clustering, time, and enrollment after COVID. Additional models controlled for demographics.

**Results:**

A total of 44% of DA arm patients reviewed the DA and 58% in UC arm reported reviewing “any information.” Being in the DA arm versus UC was not associated with knowledge. However, “review of information” was associated with higher knowledge. In addition, non‐White race and lower education were associated with lower knowledge. The DA arm was associated with higher perceptions of feeling informed (parameter estimate 1.36; 95% CI, 0.18–2.55; *p* = .02); this persisted even when controlling for review of information or demographics.

**Conclusion:**

Improved knowledge was not demonstrate with a web‐based DA versus UC. Interestingly, the DA was associated with a higher likelihood of feeling informed. Future research will explore the discrepancy between patients feeling informed but having low knowledge, especially for disadvantaged patients.

**Trial Registration:**

ClinicalTrials.gov Identifier: NCT0376600

## Abstract 2

Although in this stepped‐wedge clinical trial we did not demonstrate improved knowledge with a web‐based DA versus UC, we observed that the DA was associated with a higher likelihood of feeling informed. Future research will explore the discrepancy between patients feeling informed but having low knowledge, especially for disadvantaged patients.

## INTRODUCTION

Decision aids (DAs) support shared decision making by providing information, establishing role expectations during the consult, and increasing patients’ confidence in surgeon interactions.^1^ DAs exist in many forms (paper‐based, websites, videos), and have been designed to be used both within the consult and outside of the consult (before and/or after). Existing data do not support one mode as superior.^2^ It is likely that the optimal mode and timing of DA delivery may vary based on the clinical scenario,^1^, ^3^ as the benefits associated with DAs can only be realized if the DA reaches the “right patient at the right time.”^4^

Through discussions with patients, nurses, and surgeons, we determined that delivery of a DA before the surgical consultation would best match the informational needs of women newly diagnosed with breast cancer.^5^, ^6^ The decision for type of breast cancer surgery is highly preference‐sensitive, as survival is equivalent between mastectomy and breast‐conserving surgery but with obvious differences in quality of life. However, it is challenging for patients to engage in decision making because of the “overwhelming” emotions associated with a cancer diagnosis, the new influx of information related to the cancer, and the hierarchical differences between the patient and the surgeon.^7^ We anticipated that providing the DA after the diagnosis but before the consult might support women by addressing these challenges.

We conducted a stepped wedge clinical trial to evaluate the effect of a web‐based DA on patient engagement in decision making.^8^, ^9^ We measured knowledge of the treatment options as a secondary outcome in the trial, as patients must have knowledge of the treatment options to engage in decision making.^10^ We conducted our study within clinics that care for a high proportion of socioeconomically disadvantaged patients, as prior literature suggests that disadvantaged patients may benefit most from the use of a DA.^11^ The objective of this study was to test the effectiveness of a surgical web‐based DA in improving knowledge among patients with breast cancer in clinics that care for a high proportion of socioeconomically disadvantaged patients.

## METHODS

### Trial design

We conducted a stepped wedge trial (A231701CD) with seven waves in 10 surgical clinics within the National Cancer Institute Community Oncology Research Program from June 2019 through December 2021.^8^, ^9^ Participating clinics cared for a high proportion of socioeconomically disadvantaged patients and represented diverse geographic settings across the United States. All clinics began in the usual care (UC) arm and were randomized to the time of transition from UC to delivery of a web‐based DA. Clinic‐level randomization was stratified based on whether the site was a minority/underserved National Cancer Institute Community Oncology Research Program. The Alliance for Clinical Trials in Oncology (Alliance) was the research base for this trial and was responsible for protocol development, data collection and management, statistical analysis, and overall study operations.^12^ Details of A231701CD have been previously published.^8^ The study was approved by the National Cancer Institute Central Institutional Review Board. The Alliance Data and Safety Monitoring Board monitored the study. Data quality was ensured by review of data by the Alliance Statistics and Data Management Center and by the study chairperson (H.B.N.) following Alliance policies.

### Usual care

During UC, each site provided patient education and clinical care per their usual practice.

### Intervention

The intervention for this trial was a web‐based DA delivered before the surgical consult that is designed to help patients with early‐stage breast cancer understand surgical options and make a decision about treatment. The decision aid, developed through a partnership between Health Dialog and the Society for Medical Decision Making, contained general information about breast cancer, a discussion about the options for breast cancer surgery, and a section directly comparing the pros and cons of mastectomy versus breast conservation. The web‐based decision aid uses didactic information written for an 8th grade reading level and clinical vignette videos to promote consideration of preferences and values. It has been used successfully in academic and community settings.^13^, ^14^, ^15^, ^16^ We delivered the DA before the surgical consult based on feedback from patient, nurse, and surgeon stakeholders, who perceived that this timing best met patients’ informational needs and would prepare them to participate actively in the consult.^5^, ^6^ The process to implement the DA into clinical work flow was based on pilot work from the investigator team.^15^, ^16^ The investigator team conducted clinic site visits to support implementation at the time of transition to the DA arm. After each clinic crossed over to the intervention arm, all new patients with breast cancer being cared for within that clinic should have been offered the web‐based DA and emailed a link before the surgical consultation as a component of standard clinical care in the clinic (consent not required to be sent the decision aid). Patients who reported difficulty with internet access were offered the option of sharing the decision aid with a family or friend or reviewing it in clinic on an iPad (provided as part of the study).

### Patient eligibility and recruitment

Patients were eligible if they were female, aged ≥18 years, had newly diagnosed stage 0 through 3 breast cancer, and were planning surgery for breast cancer. Patients were excluded if they had impaired decision‐making capacity or required an interpreter. The research teams at each clinic prescreened clinic schedules and patients were approached by a research coordinator before the surgical consult to obtain informed consent.

### Data collection

**FIGURE 1:**
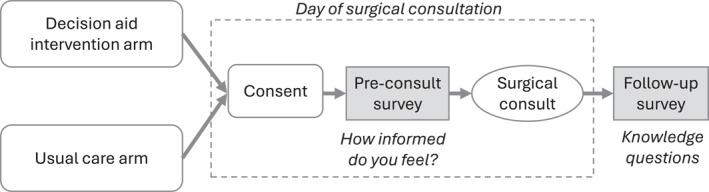
Overview of data collection.

Patients in both the UC and the DA arm completed a paper survey in clinic at the time of consent (right before surgical consultation) and a follow‐up survey (sent by email as a Qualtrics survey the day after the clinic visit) (Figure 1). Patients received an incentive of $10 for participating in the audio recording and/or completing the follow‐up survey. Data were also abstracted from the electronic medical record.

### Measures

The outcome was patient knowledge (a secondary outcome for the clinical trial). We measured patient knowledge on the follow‐up survey after the surgical consult using the Breast Cancer Surgery Decision Quality Instrument.^17^ This is a five‐item validated scale developed with the intent of assessing the extent to which patients were informed about breast cancer surgery (e.g., relative recurrence risk for lumpectomy vs mastectomy); it is reported as percent correct. We also assessed patients’ perceptions about feeling informed through a single question “how informed do you feel?” This was asked on the survey at the time of consent before the surgical consultation on a 0 to 10 scale, where 10 is “highly informed.”

The primary explanatory variable was study arm (DA vs UC). We assessed socioeconomic disadvantage using the Area Deprivation Index measured at the ZIP+4 level (2020 version) and dichotomized at the 8th decile as disadvantaged (yes/no).^18^, ^19^ We collected self‐reported education and dichotomized as having a college degree (yes/no). We also asked participants about review of information before the consult. Participants in the DA arm were asked whether they were “able to review the website sent to you before coming to see your surgeon today?” and participants in the UC arm were asked “did you review any information before coming to see your surgeon today?”

### Analysis plan

Patient characteristics were summarized overall and by study arm. The primary analyses were intention‐to‐treat without consideration of whether patients in the DA arm received or reviewed the DA. Intervention effects for each outcome was tested with linear mixed‐effects models, accounting for surgeon and clinic‐level clustering, time, and enrollment after COVID. Intraclass correlations were calculated for surgeon and clinic for each outcome. Patients were included in the analytic cohort for the knowledge outcome if they had completed the knowledge questions on the follow‐up survey. Patients were included in the analytic cohort for the feeling informed outcome if they completed the survey at time of consent. Analysis of stepped wedge studies require a model for time effects; this was complicated for our study given the COVID‐19 pandemic and associated temporary study closures. To acknowledge and account for time trends, we included additional parameters in the model (wave and wave‐squared) to flexibly control for time effects and to allow for a nonlinear relationship between time and outcome measures. In addition, we included two parameters to account specifically for COVID‐19: an indicator for whether the patient enrolled before versus after March 2020, and the wave number when the clinic resumed enrollment. Power and sample size for A231701CD clinic trial was calculated on the primary outcome of engagement (rather than the secondary outcome of knowledge described in this manuscript) and is reported elsewhere.^8^

We created additional models including key patient characteristics potentially associated with knowledge and/or feeing informed variables (age, race, college degree socioeconomic disadvantage).^11^ We conducted two exploratory analyses considering whether patients reported either reviewing the DA (DA arm) or “any information” (UC arm) before the consult. The first included the review variable as an explanatory variable models with the whole cohort, whereas the second restricted the cohort to those that reported reviewing materials prior to the consult.

## RESULTS

**FIGURE 2:**
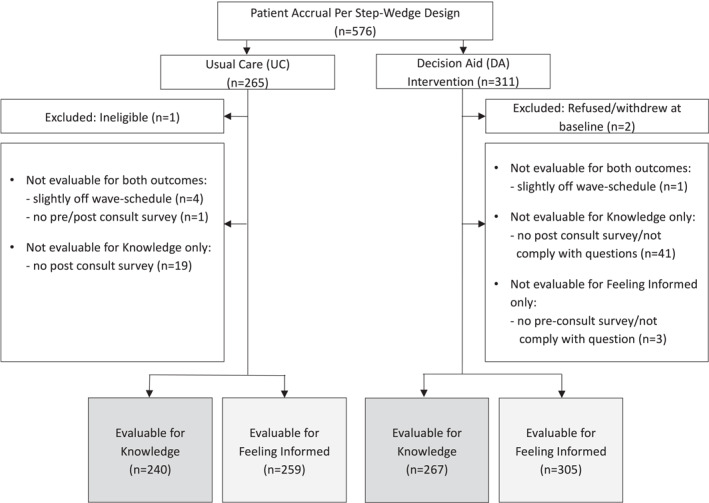
Overview of patient enrollment and eligibility for evaluation of knowledge and feeling informed outcomes.

Overall, 576 patients provided consent for the study, three of whom were excluded from the analysis (Figure 2). A total of 507 patients completed the knowledge questions on the follow‐up survey and were included in the analysis for knowledge, whereas 564 patients responded to the question about feeling informed on the survey before the consultation. Patients were a median of 60 years of age (range 27–90) and most had small, node‐negative cancers (Table 1). The cohort was racially diverse: 66% White and 21% Black. Based on the Area Deprivation Index, 23% of patients lived in an area of socioeconomic disadvantage; 36% did not have a college degree. Overall, 44% of patients in the DA intervention arm reviewed the DA and 58% of patients in the UC arm reported reviewing “any information” before the surgical consultation.

**TABLE 1:** Characteristics of participating patients.

	Total (*n* = 573)	Usual care (*n* = 264)	Decision aid (*n* = 309)
Age (years, median, range)	60 (27–90)	59 (30–87)	61 (27–90)
Race			
White	376 (66%)	169 (64%)	207 (67%)
Black	122 (21%)	60 (23%)	62 (20%)
Other	75 (13%)	35 (13%)	40 (13%)
Hispanic or Latino	27 (5%)	6 (2%)	21 (7%)
Socioeconomic disadvantage	132 (23%)	73 (28%)	59 (19%)
No college degree	206 (36%)	110 (42%)	96 (31%)
T stage			
T0	94 (16%)	40 (15%)	54 (18%)
T1/T2	411 (72%)	188 (71%)	223 (72%)
T3/T4	49 (9%)	20 (8%)	29 (9%)
Tx or missing	19 (3%)	16 (6%)	3 (1%)
Nodal status			
N0	438 (76%)	191 (72%)	247 (80%)
N1/N2/N3	84 (15%)	38 (14%)	46 (15%)
Nx or missing	51 (9%)	35 (13%)	16 (5%)

**FIGURE 3:**
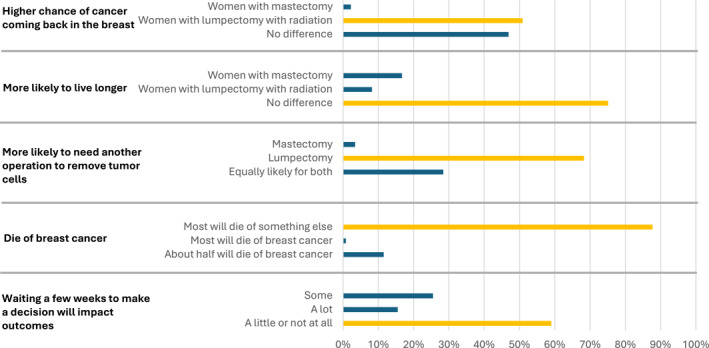
Patient responses to individual knowledge questions. As no differences were observed between the decision aid intervention arm and usual care, responses from the two study arms were combined. The response with the yellow bar represents the correct response.

The mean correct knowledge score was 69.6% (standard deviation [SD] 21.4) for patients in the DA arm and 66.1% (SD 21.8) for patients in the UC arm. Figure 3 presents the responses to individual questions, with the yellow bar marking the correct response. There was no statistical difference in the percent correct for individual questions between the UC and DA arm (Supplemental Figure 1). Patients were least likely to correctly answer questions about the risk of cancer coming back in the breast (50.9% with correct response) or the impact of waiting a few weeks to make a decision would have (58.9% correct).

Being in the DA intervention arm versus UC was not statistically associated with total knowledge score (Table 2, model 1). However, non‐White race and lower education were associated with lower total knowledge scores (model 2). In the exploratory model, patient review of information (the web‐based DA in the intervention arm or “any information” in the UC arm) was associated with higher knowledge scores (model 3).

**TABLE 2:** Impact of a web‐based decision aid on knowledge.

	Model 1 (*n* = 507)^a^	Model 2 (*n* = 504)	Exploratory model 3 (*n* = 498)^a^
Intervention status	2.74 (–5.56 to 11.03)	0.56 (–7.48 to 8.59)	0.36 (–7.54 to 8.26)
Age		–0.09 (–0.24 to 0.06)	–0.08 (–0.23 to 0.07)
Race			
White		Ref	Ref
Black		–7.53 (–12.99 to –2.07)^a^	–6.52 (–11.95 to –1.09)^a^
Other		–8.46 (–14.87 to –2.06)^a^	–7.87 (–14.23 to –1.50)^a^
No college degree		–6.34 (–10.28 to –2.40)^a^	–5.63 (–9.56 to –1.71)^a^
Socioeconomic disadvantage		–2.05 (–6.65 to 2.54)^a^	–1.11 (–5.71 to 3.48)^a^
Review of information			5.73 (1.89–9.57)

*Note*: Model 1 represents the main effect of the decision aid. Model 2 controls for key patient demographics associated with knowledge. Model 3 is an exploratory analysis that also considers whether patients reviewed information prior to the surgical consult. Model 1 within‐surgeon intraclass correlation coefficient = 0.0204, within‐site‐between surgeon = intraclass correlation coefficient 0.0026.

^a^ *p* < .05; *n* varies based on missingness of variables.

The mean score for perceptions of feeling informed was 4.8 (SD 3.2) for UC and 5.6 (SD 3.3) for DA. The DA intervention arm was associated with higher perceptions of feeling informed (parameter estimate 1.36; 95% CI, 0.18–2.55; *p* = .02) (Table 3, model 1). This persisted even after controlling for patient demographics (model 2) and whether patients reviewed “any information” before the consultation (exploratory model 3). Black race was associated with feeling informed (using White as the reference). In addition, review of information was associated with feeling informed.

**TABLE 3:** Impact of a web‐based decision aid on patient perceptions of feeling informed.

	Model 1 (*n* = 564)^a^	Model 2 (*n* = 502)^a^	Exploratory model 3 (*n* = 498)^a^
DA intervention (ref: usual care)	1.36 (0.18–2.55)^a^	1.97 (0.71–3.24)^a^	1.96 (0.71–3.21)^a^
Age		–0.001 (–0.02 to 0.02)	0.005 (–0.02 to 0.03)
Race			
White		Ref	Ref
Black		0.79 (–0.05 to 1.64)	0.99 (0.15–1.83)^a^
Other		0.73 (–0.26 to 1.72)	0.81 (–0.17 to 1.79)
No college degree		0.33 (–0.25 to 0.91)	0.35 (–0.23 to 0.93)
Socioeconomic disadvantage		0.12 (–0.57 to 0.81)	0.19 (–0.50 to 0.87)
Review of information			1.04 (0.48–1.60)^a^

*Note*: Model 1 within‐surgeon intraclass correlation coefficient = 0.007, within‐site‐between surgeon = intraclass correlation coefficient 0.0381. Model 1 represents the main effect of the decision aid. Model 2 controls for whether patients reviewed information. Model 3 additionally controls for key patient demographics associated with engagement.

^a^ *p* < .05; *n* varies based on missingness of variables.

## DISCUSSION

In this stepped wedge trial conducted in clinics that serve diverse populations across the United States, we found that patients in the web‐based DA arm were more likely to feel informed compared to patients in the UC arm, though no difference in knowledge was observed. This observation was robust, even after controlling for whether patients reviewed information and for patient demographics.

Knowledge has commonly been used to assess the effectiveness of DAs, and most studies evaluating breast cancer DAs have observed improved knowledge.^14^, ^20^, ^21^, ^22^, ^23^ The discrepancy between our study, where we did not see higher knowledge with a DA, and that of prior trials likely reflect differences in study design features. For example, in the prospective study by Belkora et al., knowledge was assessed only for patients who reviewed the DA; knowledge was assessed before and after review of the DA (51% and 74% correct, respectively).^14^ Similarly, in a recent randomized controlled trial by Hawley et al., consent for the study was obtained only after patients logged into the web‐based DA; the vast majority reviewed DA content before they were asked to complete the survey knowledge questions.^23^ In this study, 60.7% of patients reported a score exceeding 80% correct (compared with only 42.5% in the control group).

In contrast, our study was based on a more pragmatic research design that integrated DA distribution into usual clinical workflows (as opposed to relying on research staff to distribute the DA to patients). We recognized when designing the study that implementation of DAs into clinical flow is a significant barrier to DAs being able to reach the patients they are intended to help.^13^, ^24^, ^25^, ^26^, ^27^, ^28^, ^29^, ^30^, ^31^, ^32^ We had developed a package of implementation strategies to support our implementation based on significant pilot testing.^15^, ^16^ Unfortunately, the COVID‐19 pandemic significantly limited our ability to execute the strategies and clinics’ ability to prioritize implementation in the face of other, unprecedented challenges. As a result, only 44% of patients enrolled in the DA arm reviewed the DA. We found no difference in percent correct between the DA and UC arm (69.6% vs 66.1%) or in the proportion that reported a score exceeding 80% correct (18.4% vs 15.4%). When we compare our results to that seen in our pilot work, we have comparable knowledge in the UC arm (66% correct in our trial and our pilot work).^16^ However, knowledge in the intervention arm was lower in this study (70%) compared to our prior work (80%), where the vast majority of patients (85%) reviewed the DA. The lower uptake of the DA in this study makes it challenging to fully assess the effect of the DA on knowledge.

It is concerning to see the lower knowledge scores associated with minority patients and those with less education. In this study, we assessed knowledge after the surgical consultation. This means that our findings reflect not only issues with the effectiveness of the DA itself in improving knowledge but also with the information exchange during the patient–surgeon interaction. Prior research has demonstrated that patient–clinician communication varies across patient groups, specifically with regard to education, race, and age.^33^, ^34^, ^35^ Future research should focus on how differences in communication influence patients’ understanding of their disease and treatment options, critical steps to actively engaging in decision making.^36^

We found that patients in the DA arm reported feeling more informed compared to those in the UC arm. This finding is highly relevant to our conceptualization of how to support patient engagement in decision making. Our study is based on the theory that to engage in decision making, patients must have knowledge of the treatment options but also “power,” defined as the “perceived influence on the decision‐making encounter, e.g. permission to participate, confidence in own knowledge, and self‐efficacy in using shared decision making skills.”^10^ We had anticipated that a DA could prepare patients for the surgical consultation by establishing that their input is essential for the decision and hoped that this would lead to increased patient confidence in their ability to interact with the surgeon during the consultation.^8^, ^9^ Our findings that patients feel more informed is a factor that contributes to this concept of “power,” that extra confidence may allow patients to speak up when they would otherwise defer to the surgeon given the differences in hierarchy. It is also important that this effect was only minimally influenced by race, education or socioeconomic status. Although we ideally want both an increase in knowledge and power, having a simple intervention that leads to patients feeling more informed and potentially being more confident is a significant first step. Future research will explore the discrepancy between patients feeling informed but having low knowledge even after the consultation.

Our work has some limitations. First, this stepped wedge trial was administered during the COVID‐19 pandemic, which influenced enrollment, study administration, and implementation of the DA into clinical workflow. Our analysis accounts for these unexpected challenges to the extent possible but this is still a limitation. Given the relatively low rate of review of the DA in the intervention arm, we attempted to define a similar cohort in the UC arm for exploratory comparisons of a cohort of participants who reviewed information before the consult. Although this was the most comparable cohort we could identify from the UC arm, it is important to acknowledge that this is a different question than what was asked in the DA arm (“did your review the decision aid”), and the populations differ to some degree. Next, although the decision aid was designed to be accessible for patients with lower health literacy (i.e., simplified language, figures, summary tables, video vignettes), it is possible that the format of the decision aid did not meet the needs of socioeconomically disadvantaged patients. Finally, we did not standardize patient education while each site was in the usual care arm. Consequently, there is the potential for variation between sites; this may have influenced our ability to detect an impact of the decision aid on knowledge.

In conclusion, this stepped wedge clinical trial evaluating a web‐based DA did not demonstrate a difference in knowledge between the DA arm compared with UC. However, we demonstrated that non‐White patients, those with lower education, and those who were socioeconomically disadvantaged had lower knowledge. Interestingly, receipt of the DA was associated with a higher likelihood of reporting feeling informed. Future research will explore the discrepancy between patients feeling informed but having low knowledge, especially for historically disadvantaged patient populations.

## Author contributions

**Jessica R. Schumacher**: Conceptualization; funding acquisition; methodology; writing—review and editing. **Bret M. Hanlon**: Writing—review and editing; formal analysis; methodology; conceptualization. **David Zahrieh**: Writing—review and editing; formal analysis; methodology; data curation. **Paul J. Rathouz**: Conceptualization; methodology; funding acquisition; writing—review and editing; formal analysis; supervision. **Jennifer L. Tucholka**: Writing—review and editing; project administration; conceptualization. **Grace McKinney**: Project administration; writing—review and editing. **Angelina D. Tan**: Formal analysis; writing—review and editing; methodology; data curation. **Catherine R. Breuer**: Writing—review and editing; project administration. **Lisa Bailey**: Investigation; writing—review and editing. **Anna M. Higham**: Investigation; writing—review and editing. **Julie S. Wecsler**: Investigation; writing—review and editing. **Alicia Arnold**: Investigation; writing—review and editing. **Anthony J. Froix**: Investigation; writing—review and editing. **Scott Dull**: Investigation; writing—review and editing. **Andrea M. Abbott**: Investigation; writing—review and editing. **Stephanie G. Fine**: Investigation; writing—review and editing. **Kandace P. McGuire**: Investigation; writing—review and editing. **Anna S. Seydel**: Investigation; writing—review and editing. **Patricia McNamara**: Project administration; data curation; writing—review and editing. **Selina Chow**: Resources; supervision; data curation; project administration; writing—review and editing. **Heather B. Neuman**: Conceptualization; investigation; writing—original draft; funding acquisition; writing—review and editing; supervision.

## CONFLICT OF INTEREST STATEMENT

Authors have no conflicts of interest relevant to the research presented within this manuscript.

## Supporting information

Supplementary Material

## Data Availability

Data will be available by request per the policy of the Alliance for Clinical Trials in Oncology (https://www.allianceforclinicaltrialsinoncology.org).

## References

[1] Stacey D , Lewis KB , Smith M , et al. Decision aids for people facing health treatment or screening decisions. Cochrane Database Syst Rev. 2024;1:CD001431. doi:10.1002/14651858.CD001431.pub6 38284415 PMC10823577

[2] Salwei ME , Ancker JS , Weinger MB . The decision aid is the easy part: workflow challenges of shared decision making in cancer care. JNCI. 2023;115(11):1271‐1277. doi:10.1093/jnci/djad133 37421403 PMC13389708

[3] GaMVM E . Tools to engage patients in clinical encounters. In: Glyn EAE , Thompson R , eds. Shared Decision Making in Health Care: Achieving evidence‐based patient choice. 3rd ed. Oxford University Press; 2016.

[4] Hoffman AS , Volk RJ , Saarimaki A , et al. Delivering patient decision aids on the internet: definitions, theories, current evidence, and emerging research areas. BMC Med Inf Decis Making. 2013;13(Suppl 2):S13. doi:10.1186/1472-6947-13-s2-s13 PMC4043476 24625064

[5] Belkora JK , Miller MF , Dougherty K , Gayer C , Golant M , Buzaglo JS . The need for decision and communication aids: a survey of breast cancer survivors. J Commun Support Oncol. 2015;13(3):104‐112. doi:10.12788/jcso.0116 25880673

[6] Hack TF , Degner LF , Parker PA . The communication goals and needs of cancer patients: a review. Psychooncology. 2005;14(10):831‐845. discussion 846‐837. doi:10.1002/pon.949 16200519

[7] Saucke MC , Jacobson N , McKinney G , Neuman HB . Role of the surgeon in de‐escalating emotion during a breast cancer surgery consultation: a qualitative study of patients' experiences in Alliance A231701CD. Ann Surg Oncol. 2024;31(13):8873‐8881. doi:10.1245/s10434-024-16156-1 39320397 PMC11803603

[8] Schumacher JR , Zahrieh D , Chow S , et al. Increasing socioeconomically disadvantaged patients' engagement in breast cancer surgery decision‐making through a shared decision‐making intervention (A231701CD): protocol for a cluster randomised clinical trial. BMJ Open. 2022;12(11):e063895. doi:10.1136/bmjopen-2022-063895 PMC9677005 36396308

[9] Schumacher JR , Hanlon BM , Zahrieh D , et al. Impact of a web‐based decision aid on socioeconomically disadvantaged patients' engagement in breast surgery decision‐making: Stepped‐Wedge Clinical Trial (Alliance‐A231701CD). Ann Surg Oncol. 2025;32(8):5540‐5550. doi:10.1245/s10434-025-17452-0 40382452 PMC12222412

[10] Joseph‐Williams N , Elwyn G , Edwards A . Knowledge is not power for patients: a systematic review and thematic synthesis of patient‐reported barriers and facilitators to shared decision making. Patient Educ Counsel. 2014;94(3):291‐309. doi:10.1016/j.pec.2013.10.031 24305642

[11] Durand MA , Carpenter L , Dolan H , et al. Do interventions designed to support shared decision‐making reduce health inequalities? A systematic review and meta‐analysis. PLoS One. 2014;9(4):e94670. doi:10.1371/journal.pone.0094670 24736389 PMC3988077

[12] Earle CC , Neville BA . Under use of necessary care among cancer survivors. Cancer. 2004;101(8):1712‐1719. doi:10.1002/cncr.20560 15386307

[13] Legare F , Ratte S , Gravel K , Graham ID . Barriers and facilitators to implementing shared decision‐making in clinical practice: update of a systematic review of health professionals' perceptions. Patient Educ Counsel. 2008;73(3):526‐535. doi:10.1016/j.pec.2008.07.018 18752915

[14] Belkora JK , Volz S , Teng AE , Moore DH , Loth MK , Sepucha KR . Impact of decision aids in a sustained implementation at a breast care center. Patient Educ Counsel. 2012;86(2):195‐204. doi:10.1016/j.pec.2011.05.011 21665420

[15] Bruce JG , Tucholka JL , Steffens NM , Mahoney JE , Neuman HB . Feasibility of providing web‐based information to breast cancer patients prior to a surgical consult. J Cancer Educ. 2017. In press. 10.1007/s13187-017-1207-6 PMC5623157 28361360

[16] Tucholka JL , Yang D , Bruce JG , et al. A randomized controlled trial evaluating the impact of web‐based information on breast cancer patient's knowledge of surgical treatment. J Am Coll Surg. 2017. in press. 10.1016/j.jamcollsurg.2017.10.024 PMC6295317 29246705

[17] Lee CN , Chang Y , Adimorah N , et al. Decision making about surgery for early‐stage breast cancer. J Am Coll Surg. 2012;214(1):1‐10. doi:10.1016/j.jamcollsurg.2011.09.017 22056355 PMC3256735

[18] Kind AJ , Jencks S , Brock J , et al. Neighborhood socioeconomic disadvantage and 30‐day rehospitalization: a retrospective cohort study. Ann Intern Med. 2014;161(11):765‐774. doi:10.7326/m13-2946 25437404 PMC4251560

[19] Singh GK . Area deprivation and widening inequalities in US mortality, 1969‐1998. Am J Public Health. 2003;93(7):1137‐1143. doi:10.2105/ajph.93.7.1137 12835199 PMC1447923

[20] Trikalinos TA , Wieland LS , Adam GP , Zgodic A , Ntzani EE . AHRQ Comparative Effectiveness Reviews. In: Decision Aids for Cancer Screening and Treatment. Agency for Healthcare Research and Quality (US); 2014. 25632492

[21] Waljee JF , Rogers MA , Alderman AK . Decision aids and breast cancer: do they influence choice for surgery and knowledge of treatment options? J Clin Oncol. 2007;25(9):1067‐1073. doi:10.1200/jco.2006.08.5472 17369570

[22] Tucholka JL , Yang DY , Bruce JG , et al. A randomized controlled trial evaluating the impact of web‐based information on breast cancer patients' knowledge of surgical treatment options. J Am Coll Surg. 2017. 10.1016/j.jamcollsurg.2017.10.024 PMC6295317 29246705

[23] Hawley ST , Li Y , An LC , et al. Improving breast cancer surgical treatment decision making: the iCanDecide Randomized Clinical Trial. J Clin Oncol. 2018;36(7):659‐666. doi:10.1200/jco.2017.74.8442 29364772 PMC5946719

[24] Sinha G . Decision aids help patients but still are not widely used. JNCI. 2014;106(7). doi:10.1093/jnci/dju224 25006197

[25] Elwyn G , Scholl I , Tietbohl C , et al. Many miles to go”: a systematic review of the implementation of patient decision support interventions into routine clinical practice. BMC Med Inf Decis Making. 2013;13(Suppl 2):S14. doi:10.1186/1472-6947-13-s2-s14 PMC4044318 24625083

[26] O'Donnell S , Cranney A , Jacobsen MJ , Graham ID , O'Connor AM , Tugwell P . Understanding and overcoming the barriers of implementing patient decision aids in clinical practice. J Eval Clin Pract. 2006;12(2):174‐181. doi:10.1111/j.1365-2753.2006.00613.x 16579826

[27] Silvia KA , Ozanne EM , Sepucha KR . Implementing breast cancer decision aids in community sites: barriers and resources. Health Expect. 2008;11(1):46‐53. doi:10.1111/j.1369-7625.2007.00477.x 18275401 PMC5060426

[28] Silvia KA , Sepucha KR . Decision aids in routine practice: lessons from the breast cancer initiative. Health Expect. 2006;9(3):255‐264. doi:10.1111/j.1369-7625.2006.00393.x 16911140 PMC5060360

[29] Holmes‐Rovner M , Valade D , Orlowski C , Draus C , Nabozny‐Valerio B , Keiser S . Implementing shared decision‐making in routine practice: barriers and opportunities. Health Expect. 2000;3(3):182‐191. 11281928 10.1046/j.1369-6513.2000.00093.x PMC5080967

[30] Charles C , Gafni A , Whelan T . Self‐reported use of shared decision‐making among breast cancer specialists and perceived barriers and facilitators to implementing this approach. Health Expect. 2004;7(4):338‐348. doi:10.1111/j.1369-7625.2004.00299.x 15544686 PMC5060255

[31] Feibelmann S , Yang TS , Uzogara EE , Sepucha K . What does it take to have sustained use of decision aids? A programme evaluation for the Breast Cancer Initiative. Health Expect. 2011;14(Suppl 1):85‐95. doi:10.1111/j.1369-7625.2010.00640.x 21323821 PMC5057173

[32] Elwyn G , Rix A , Holt T , Jones D . Why do clinicians not refer patients to online decision support tools? Interviews with front line clinics in the NHS. BMJ Open. 2012;2(6):e001530. doi:10.1136/bmjopen-2012-001530 PMC3532981 23204075

[33] Street RL, Jr . Information‐giving in medical consultations: the influence of patients' communicative styles and personal characteristics. Soc Sci Med. 1991;32(5):541‐548. doi:10.1016/0277-9536(91)90288-n 2017721

[34] Waitzkin H . Information giving in medical care. J Health Soc Behav. 1985;26(2):81‐101. doi:10.2307/2136599 4031436

[35] Epstein RMSRLJ . Patient‐Centered Communication in Cancer Care: Promoting Healing and Reducing Suffering. National Cancer Institute; 2007. Vol NIH Pulbication No. 07‐6225.

[36] Gilligan T , Coyle N , Frankel RM , et al. Patient‐clinician communication: American Society of Clinical Oncology Consensus Guideline. J Clin Oncol. 2017;35(31):3618‐3632. doi:10.1200/jco.2017.75.2311 28892432

