# Supplementary figures and images for: Impact of a web‐based breast cancer surgery decision aid on knowledge and perceptions of feeling informed in clinics that care for socioeconomically disadvantaged patients: An Alliance Clinical Trial (A231701CD)
Source: Cancer. 2026 Feb 11;132(4):e70314. doi: 10.1002/cncr.70314 (PMC12892235; doi:10.1002/cncr.70314)

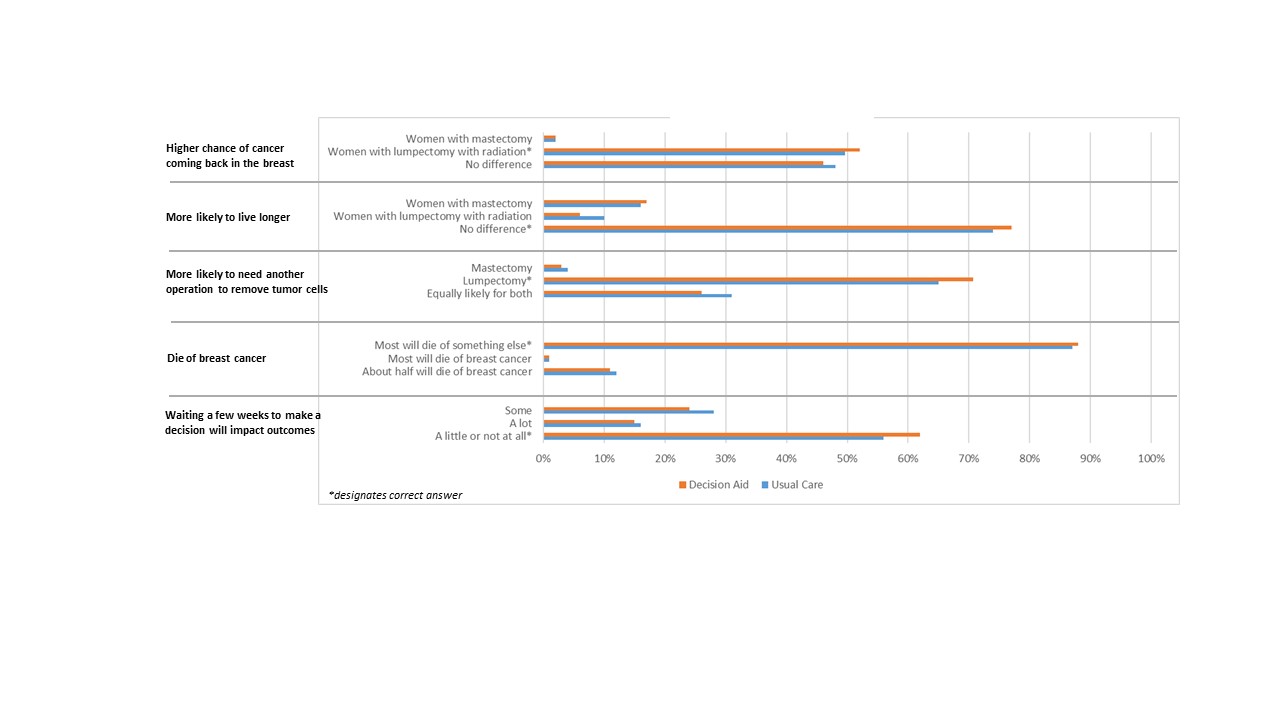

Supplement: Supplementary file 1 — Supplementary Material [file CNCR-132-e70314-s001.jpg]
